# Supplementary material for: Subcellular analysis of pigeon hair cells implicates vesicular trafficking in cuticulosome formation and maintenance
Source: eLife. 2017 Nov 15;6:e29959. doi: 10.7554/eLife.29959 (PMC5699870; doi:10.7554/eLife.29959)
Supplement: Figure 1—source data 1. — This table shows the estimated numbers of total hair cells, total PB +ve hair cells and the percentage of PB +ve hair cells in the basilar papilla and lagenar macula of all pigeons used for the analysis shown in Figure 1c and d and Figure 1—figure supplements 2–6 ‘R’ and”L’ stand for ‘right’ or ‘left’ cochlear duct. [file elife-29959-fig1-data1.docx]

| **Bird** | **Age** | **Basilar papilla** | | | **Lagenar macula** | | |
| --- | --- | --- | --- | --- | --- | --- | --- |
|  |  | **Estimated total hair cell number** | **Estimated PB+ hair cell number** | **% of hair cells that are PB+ve** | **Estimated total hair cell number** | **Estimated PB+ hair cell number** | **% of hair cells that are PB+ve** |
| A63R | 1 day | 9424 | 464 | 4.9 | 5024 | 4 | 0.08 |
| A65L | 1 day | 6016 | 84 | 1.4 | 4772 | 0 | 0 |
| A66L | 1 day | 9920 | 64 | 0.64 | 5752 | 4 | 0.07 |
| A107R | 1 day | 11284 | 332 | 2.9 | 9340 | 8 | 0.09 |
| **Average** |  | 9161 | 236 | 2.46 | 6222 | 4 | 0.06 |
| **SEM** |  | 1119.65 | 97.42 | 0.95 | 1059.89 | 1.63 | 0.019 |
|  |  |  |  |  |  |  |  |
| A80R | 8 days | 12808 | 2252 | 17.6 | 9492 | 20 | 0.21 |
| A211L | 8 days | 13300 | 4044 | 30.4 | 8652 | 36 | 0.42 |
| A212L | 8 days | 11184 | 2572 | 23 | 7252 | 80 | 1.10 |
| **Average** |  | 12430.67 | 2956 | 23.66 | 8465.3 | 45.33 | 0.57 |
| **SEM** |  | 521.99 | 450.53 | 3.03 | 533.44 | 14.65 | 0.22 |
|  |  |  |  |  |  |  |  |
| A113R | 16 days | 13036 | 2880 | 22.09 | 15896 | 220 | 1.38 |
| A114L | 16 days | 15092 | 4472 | 29.63 | 16192 | 212 | 1.31 |
| A115R | 16 days | 15820 | 4424 | 27.96 | 17784 | 144 | 0.81 |
| A116R | 16 days | 16004 | 5716 | 35.72 | 17684 | 80 | 0.45 |
| A117L | 16 days | 15812 | 4640 | 29.34 | 19276 | 100 | 0.52 |
| A118L | 16 days | 10794 | 4575 | 42.38 | 16364 | 200 | 1.22 |
| **Average** |  | 14426.33 | 4451.17 | 31.19 | 17199.33 | 159.33 | 0.95 |
| **SEM** |  | 854.82 | 370.61 | 2.86 | 526.01 | 24.60 | 0.17 |
|  |  |  |  |  |  |  |  |
| A83L | 28-30 days | 14528 | 4928 | 33.92 | 19324 | 104 | 0.54 |
| A84L | 28-30 days | 14880 | 4616 | 31.02 | 17032 | 160 | 0.94 |
| A85R | 28-30 days | 11824 | 4556 | 38.53 | 12312 | 260 | 2.11 |
| A103 | 28-30 days | 11312 | 3512 | 31.05 | 15736 | 240 | 1.53 |
| A108R | 28-30 days | 12124 | 6068 | 50.05 | 15132 | 524 | 3.46 |
| **Average** |  | 12933.6 | 3946.67 | 36.9 | 15907.2 | 214.67 | 1.72 |
| **SEM** |  | 736.44 | 409.64 | 3.56 | 1150.97 | 72.25 | 0.51 |
|  |  |  |  |  |  |  |  |
| A68R | 1 year | 9788 | 3740 | 38.21 | 19340 | 620 | 3.21 |
| A89L | 1 year | 13712 | 2248 | 16.39 | 17740 | 84 | 0.47 |
| A67R | 1 year | 9812 | 1976 | 20.14 | 20184 | 1332 | 6.59 |
| **Average** |  | 11104 | 2654.67 | 24.92 | 19088 | 678.67 | 3.43 |
| **SEM** |  | 1304.02 | 548.32 | 6.74 | 716.68 | 361.46 | 1.77 |

**Figure 1 - source data 1. Quantitation of Prussian blue positive hair cells in the basilar papilla and lagena macula.** This table shows the estimated numbers of total hair cells, total PB+ve hair cells and the percentage of PB+ve hair cells in the basilar papilla and lagenar macula of all pigeons used for the analysis shown in Figure 1c, 1d and Fig. 1-figure supplement 3-7. “R” and ”L” stand for “right” or “left” cochlear duct
