# Supplementary material for: Subcellular analysis of pigeon hair cells implicates vesicular trafficking in cuticulosome formation and maintenance
Source: eLife. 2017 Nov 15;6:e29959. doi: 10.7554/eLife.29959 (PMC5699870; doi:10.7554/eLife.29959)
Supplement: Figure 2—source data 1. — This table shows the percent of cuticulosomes that are surrounded by membranes, associated with vesicles, have a paracrystalline organisation, and those that are incomplete at 1 day, 8 days, 16 days, 30 days and 1 year. Data for 1 year was taken from (Lauwers et al., 2013). [file elife-29959-fig2-data1.docx]

|  | **1 day**  n=4 cuticulosomes  n=3 birds | **8 days**  n=26 cuticulosomes  n=3 birds | **16 days**  n=45 cuticulosomes  n=3 birds | **30 days**  n=17 cuticulosomes  n=3 birds | **1 year**  n=18 cuticulosomes  n=3 birds |
| --- | --- | --- | --- | --- | --- |
| **Membranes** | 25% | 23.08% | 15.56% | 17.64% | 22% |
| **Vesicles** | 100% | 46.15% | 24.44% | 52.94% | 25% |
| **Ordered** | 0% | 19.23% | 17.78% | 17.64% | 16% |
| **Incomplete** | 100% | 34.62% | 22.22% | 17.64% | 7% |

**Figure 2- source data 1. Properties of cuticulosomes at different ages.** This table shows the percent of cuticulosomes that are surrounded by membranes, associated with vesicles, have a paracrystalline organisation, and those that are incomplete at 1 day, 8 days, 16 days, 30 days and 1 year. Data for 1 year was taken from ([Lauwers, Pichler et al. 2013](#_ENREF_39)).
