# Supplementary material for: Subcellular analysis of pigeon hair cells implicates vesicular trafficking in cuticulosome formation and maintenance
Source: eLife. 2017 Nov 15;6:e29959. doi: 10.7554/eLife.29959 (PMC5699870; doi:10.7554/eLife.29959)
Supplement: Figure 5—source data 2. — This table shows a list of adjusted p-values, log2-fold changes and general information about the genes that were significantly, differentially expressed between hair cells with and without cuticulosomesomes. [file elife-29959-fig5-data2.docx]

| **Gene** | **Gene Information** | **Log-2 fold change**  **Cut.pos./Cut.neg.** | **p-value (adjusted)** |
| --- | --- | --- | --- |
| Transcription factor Sp4 | Member of the Sp1-family of zinc finger transcription factors, highly expressed in the developing murine nervous system and the adult brain and required for normal murine development ([Supp, Witte et al. 1996](#_ENREF_29)). Transcriptional activator that is activated by Sp1 and repressed by Sp3 in a dose dependent manner ([Hagen, Dennig et al. 1995](#_ENREF_13)). | 5.264689 | 4.50E^-06^ |
| Tumor necrosis factor receptor superfamily member 26 (Tnfrsf26) | Member of the Tumor necrosis factor receptor superfamily which are transmembrane receptors involved in cell growth, differentiation, immune responses and apoptosis ([MacEwan 2002](#_ENREF_23)). Tnfrsf26 is expressed during mouse embryogenesis and in neonatal heart and liver. The exact function of Tnfrsf26 is currently unknown ([Esperón, Cordier et al. 2012](#_ENREF_9)). | 5.529302 | 2.29E^-05^ |
| Ras-related protein Rab-5B | Family member of the Rab GTPase proteins that are involved in several steps of vesicular trafficking. Rab5b in particular is involved in mediating early steps of endocytosis, is localized to membranes of early endosomes and directly interacts with EEA1 which is an essential protein for early endosomal membrane fusion ([Wilson and Wilson 1992](#_ENREF_35), [Callaghan, Nixon et al. 1999](#_ENREF_6)). | 1.468476 | 4.18E^-05^ |
| Protein of unknown function (Cliv_009848) | Unknown | 5.201516 | 0.000491 |
| Steroid hormone receptor ERR2 (Esrrb) | Mutations in the nuclear hormone receptor Esrrb cause autosomal-recessive nonsyndromic hearing impairment in humans ([Collin, Kalay et al. 2008](#_ENREF_7)). Esrrb mediates reprogramming of mouse embryonic fibroblasts into induced pluripotent stem cells together with Oct4 and Sox2 ([Feng, Jiang et al. 2009](#_ENREF_11)), suppresses differentiation and sustains embryonic stem cell self-renewal ([Martello, Sugimoto et al. 2012](#_ENREF_24)). | 3.321123 | 0.007978 |
| Inactive carboxypeptidase-like protein X2 (CPXM2) | Metallocarboxypeptidase that is expressed in mouse brain, liver, kidney and lung ([XIN, DAY et al. 1998](#_ENREF_36)) and has been implicated in cognitive decline in schizophrenia patients ([Hashimoto, Ikeda et al. 2013](#_ENREF_14)) and in inner ear defects and deafness ([Somma, Alger et al. 2012](#_ENREF_28)). | 3.610002 | 0.014618 |
| DNA polymerase epsilon subunit 4 (Pole4) | A histone fold subunit of DNA polymerase epsilon involved in DNA transcription and repair ([Li, Pursell et al. 2000](#_ENREF_22)). | 4.302804 | 0.030966 |
| p53 apoptosis effector related to PMP-22 (PERP) | A tetraspan membrane protein involved in assembly of desmosomal adhesive complexes and epithelial integrity in a p63 dependent manner ([Ihrie, Marques et al. 2005](#_ENREF_15)) and is an apoptosis associated target of p53 ([Ihrie, Reczek et al. 2003](#_ENREF_16)). | 4.262094 | 0.030966 |
| Layilin (LAYN) | Transmembrane hyaluronan receptor that co-localizes with talin in motile membrane ruffles and suggested to be involved in reorganization of the cytoskeleton ([Borowsky and Hynes 1998](#_ENREF_4), [Bono, Rubin et al. 2001](#_ENREF_3)). | 3.919895 | 0.030966 |
| Syntaxin-binding protein 5-like (Stxbp5l) | Shares high sequence homology with Stxbp5 which is regulating syntaxin-1 mediated membrane fusion and neurotransmitter release ([Zhu, Yamakuchi et al. 2014](#_ENREF_39)), is implicated in an autosomal recessive infantile onset neurodegenerative disorder and skin aging ([Le Clerc, Taing et al. 2013](#_ENREF_21), [Kumar, Corbett et al. 2015](#_ENREF_20)). | 3.483331 | 0.030966 |
| Protein phosphatase Slingshot homolog 2 (SSH2) | Member of the F-actin binding slingshot phosphatases which are involved in actin dynamics by dephosphorylation of cofilin ([Niwa, Nagata-Ohashi et al. 2002](#_ENREF_25)). | 3.363531 | 0.030966 |
| G-protein coupled receptor 56 (Gpr56) | Transmembrane protein involved in cell matrix adhesion and regulation of brain development ([Koirala, Jin et al. 2009](#_ENREF_19)). | 3.930622 | 0.032913 |
| Zinc finger protein 385D (Znf385d) | Implicated in commonly comorbid language disorders ([Eicher 2014](#_ENREF_8)). | 2.765318 | 0.036168 |
| Heme-binding protein 2 (Hebp2) | Member of the heme binding protein family initially identified in chicken retina and pineal gland that may play a role in heme biosynthesis and uptake ([Taketani, Adachi et al. 1998](#_ENREF_30), [Zylka and Reppert 1999](#_ENREF_40)) | 2.596865 | 0.036506 |
| Ectonucleoside triphosphate diphosphohydrolase 8 (ENTPD8) | Cell surface located NTPDase that catalyzes the hydrolysis of extracellular nucleotides ([Bigonnesse, Lévesque et al. 2004](#_ENREF_2)) | 3.548147 | 0.040336 |
| Phosphodiesterase 8A (PDE8A) | Involved in the hydrolysis of the second messenger cAMP and in the regulation of RAF1-dependent EGF-activated ERK-signaling ([Wang, Yan et al. 2008](#_ENREF_34), [Brown, Day et al. 2013](#_ENREF_5)) | 3.394331 | 0.043812 |
| Quinone oxidoreductase PIG3 (TP53I3) | Possible involvement in the generation of reactive oxygen species ([Porté, Valencia et al. 2009](#_ENREF_26)). | 2.994202 | 0.043812 |
| Protein of unknown function (CLIV_018774) | Unknown | 3.161477 | 0.045818 |
| Forkhead box protein P1 (FoxP1) | Transcriptional repressor that has possible implication in speech disorders and is a transcriptional regulator of B-cell development ([Teramitsu, Kudo et al. 2004](#_ENREF_31), [van Keimpema, Grüneberg et al. 2014](#_ENREF_33)). | -3.1918198 | 2.29E^-05^ |
| Arachidonate 5-lipoxygenase (Alox5) | Involved in leukotriene biosynthesis and inflammatory processes ([Gilbert, Bartlett et al. 2011](#_ENREF_12)). | -4.0039293 | 0.02250969 |
| E3 ubiquitin-protein ligase RNF128 | Members of this protein family are involved in endocytosis and have been associated with the formation of enlarged Rab5 positive endosomes ([Yamazaki, Schonherr et al. 2013](#_ENREF_38)). | -0.8210262 | 0.02938124 |
| Leucine-rich repeat-containing protein 3B (LRRC3B) | Identified as a putative tumor suppressor gene in gastric cancer ([Kim, Kim et al. 2008](#_ENREF_18)). | -3.9681559 | 0.03096639 |
| Glycogenin-1  (Gyg1) | Involved in glycogen metabolism and implicated in glycogen storage diseases ([Fanin, Torella et al. 2015](#_ENREF_10), [Akman, Aykit et al. 2016](#_ENREF_1)). | -0.8546099 | 0.03291268 |
| Oxysterol-binding protein-related protein 2  (Osbpl2) | Involved in cellular lipid homeostasis, sterol signaling and vesicular trafficking ([Raychaudhuri and Prinz 2010](#_ENREF_27)). Specifically expressed in the stereocilia of hair cells and implicated in autosomal dominant hearing loss and progressive non syndromic hearing loss ([Xing, Yao et al. 2014](#_ENREF_37), [Thoenes, Zimmermann et al. 2015](#_ENREF_32)). | -2.1597405 | 0.03608358 |
| Probable exonuclease mut-7 homolog (EXD3) | Mut-7 is involved in transposon silencing and RNA interference in *C. elegans* ([Ketting, Haverkamp et al. 1999](#_ENREF_17)) | -2.5935553 | 0.04049373 |

**Refences for Figure 5 Source data 2.**

Akman, H. O., Y. Aykit, O. C. Amuk, E. Malfatti, N. B. Romero, M. A. Maioli, R. Piras, S. DiMauro and G. Marrosu (2016). "Late-onset polyglucosan body myopathy in five patients with a homozygous mutation in GYG1." Neuromuscular Disorders **26**(1): 16-20.

Bigonnesse, F., S. A. Lévesque, F. Kukulski, J. Lecka, S. C. Robson, M. J. Fernandes and J. Sévigny (2004). "Cloning and Characterization of Mouse Nucleoside Triphosphate Diphosphohydrolase-8†." Biochemistry **43**(18): 5511-5519.

Bono, P., K. Rubin, J. M. Higgins and R. O. Hynes (2001). "Layilin, a novel integral membrane protein, is a hyaluronan receptor." Molecular biology of the cell **12**(4): 891-900.

Borowsky, M. L. and R. O. Hynes (1998). "Layilin, a novel talin-binding transmembrane protein homologous with C-type lectins, is localized in membrane ruffles." The Journal of cell biology **143**(2): 429-442.

Brown, K. M., J. P. Day, E. Huston, B. Zimmermann, K. Hampel, F. Christian, D. Romano, S. Terhzaz, L. C. Lee and M. J. Willis (2013). "Phosphodiesterase-8A binds to and regulates Raf-1 kinase." Proceedings of the National Academy of Sciences **110**(16): E1533-E1542.

Callaghan, J., S. Nixon, C. Bucci, B. H. Toh and H. Stenmark (1999). "Direct interaction of EEA1 with Rab5b." European journal of biochemistry **265**(1): 361-366.

Collin, R. W., E. Kalay, M. Tariq, T. Peters, B. van der Zwaag, H. Venselaar, J. Oostrik, K. Lee, Z. M. Ahmed and R. Çaylan (2008). "Mutations of ESRRB encoding estrogen-related receptor beta cause autosomal-recessive nonsyndromic hearing impairment DFNB35." The American Journal of Human Genetics **82**(1): 125-138.

Eicher, J. D. (2014). Examining the Genetic Underpinnings of Commonly Comorbid Language Disorders, Yale University.

Esperón, E. d. l. C., G. Cordier and N. Engel (2012). "A genomic reservoir for tnfrsf genes is developmentally regulated and imprinted in the mouse." Epigenetics **7**(6): 626-634.

Fanin, M., A. Torella, M. Savarese, V. Nigro and C. Angelini (2015). "GYG1 gene mutations in a family with polyglucosan body myopathy." Neurology Genetics **1**(3): e21.

Feng, B., J. Jiang, P. Kraus, J.-H. Ng, J.-C. D. Heng, Y.-S. Chan, L.-P. Yaw, W. Zhang, Y.-H. Loh and J. Han (2009). "Reprogramming of fibroblasts into induced pluripotent stem cells with orphan nuclear receptor Esrrb." Nature cell biology **11**(2): 197-203.

Gilbert, N. C., S. G. Bartlett, M. T. Waight, D. B. Neau, W. E. Boeglin, A. R. Brash and M. E. Newcomer (2011). "The structure of human 5-lipoxygenase." Science **331**(6014): 217-219.

Hagen, G., J. Dennig, A. Preiß, M. Beato and G. Suske (1995). "Functional analyses of the transcription factor Sp4 reveal properties distinct from Sp1 and Sp3." Journal of Biological Chemistry **270**(42): 24989-24994.

Hashimoto, R., M. Ikeda, K. Ohi, Y. Yasuda, H. Yamamori, M. Fukumoto, S. Umeda-Yano, D. Dickinson, B. Aleksic and M. Iwase (2013). "Genome-wide association study of cognitive decline in schizophrenia." American Journal of Psychiatry **170**(6): 683-684.

Ihrie, R. A., M. R. Marques, B. T. Nguyen, J. S. Horner, C. Papazoglu, R. T. Bronson, A. A. Mills and L. D. Attardi (2005). "Perp is a p63-regulated gene essential for epithelial integrity." Cell **120**(6): 843-856.

Ihrie, R. A., E. Reczek, J. S. Horner, L. Khachatrian, J. Sage, T. Jacks and L. D. Attardi (2003). "Perp is a mediator of p53-dependent apoptosis in diverse cell types." Current biology **13**(22): 1985-1990.

Ketting, R. F., T. H. Haverkamp, H. G. van Luenen and R. H. Plasterk (1999). "Mut-7 of C. elegans, required for transposon silencing and RNA interference, is a homolog of Werner syndrome helicase and RNaseD." Cell **99**(2): 133-141.

Kim, M., J.-H. Kim, H.-R. Jang, H.-M. Kim, C.-W. Lee, S.-M. Noh, K.-S. Song, J.-S. Cho, H.-Y. Jeong and Y. Hahn (2008). "LRRC3B, encoding a leucine-rich repeat-containing protein, is a putative tumor suppressor gene in gastric cancer." Cancer research **68**(17): 7147-7155.

Koirala, S., Z. Jin, X. Piao and G. Corfas (2009). "GPR56-regulated granule cell adhesion is essential for rostral cerebellar development." Journal of Neuroscience **29**(23): 7439-7449.

Kumar, R., M. A. Corbett, N. J. Smith, L. A. Jolly, C. Tan, D. J. Keating, M. D. Duffield, T. Utsumi, K. Moriya and K. R. Smith (2015). "Homozygous mutation of STXBP5L explains an autosomal recessive infantile-onset neurodegenerative disorder." Human molecular genetics **24**(7): 2000-2010.

Le Clerc, S., L. Taing, K. Ezzedine, J. Latreille, O. Delaneau, T. Labib, C. Coulonges, A. Bernard, S. Melak and W. Carpentier (2013). "A genome-wide association study in Caucasian women points out a putative role of the STXBP5L gene in facial photoaging." Journal of Investigative Dermatology **133**(4): 929-935.

Li, Y., Z. F. Pursell and S. Linn (2000). "Identification and cloning of two histone fold motif-containing subunits of HeLa DNA polymerase ε." Journal of Biological Chemistry **275**(30): 23247-23252.

MacEwan, D. J. (2002). "TNF receptor subtype signalling: differences and cellular consequences." Cellular signalling **14**(6): 477-492.

Martello, G., T. Sugimoto, E. Diamanti, A. Joshi, R. Hannah, S. Ohtsuka, B. Göttgens, H. Niwa and A. Smith (2012). "Esrrb is a pivotal target of the Gsk3/Tcf3 axis regulating embryonic stem cell self-renewal." Cell stem cell **11**(4): 491-504.

Niwa, R., K. Nagata-Ohashi, M. Takeichi, K. Mizuno and T. Uemura (2002). "Control of actin reorganization by Slingshot, a family of phosphatases that dephosphorylate ADF/cofilin." Cell **108**(2): 233-246.

Porté, S., E. Valencia, E. A. Yakovtseva, E. Borràs, N. Shafqat, J. É. Debreczeny, A. C. Pike, U. Oppermann, J. Farrés and I. Fita (2009). "Three-dimensional structure and enzymatic function of proapoptotic human p53-inducible quinone oxidoreductase PIG3." Journal of Biological Chemistry **284**(25): 17194-17205.

Raychaudhuri, S. and W. A. Prinz (2010). "The diverse functions of oxysterol-binding proteins." Annual review of cell and developmental biology **26**: 157-177.

Somma, G., H. M. Alger, R. M. McGuire, J. D. Kretlow, F. R. Ruiz, S. A. Yatsenko, P. Stankiewicz, W. Harrison, E. Funk and A. Bergamaschi (2012). "Head bobber: an insertional mutation causes inner ear defects, hyperactive circling, and deafness." Journal of the Association for Research in Otolaryngology **13**(3): 335-349.

Supp, D. M., D. P. Witte, W. W. Branford, E. P. Smith and S. S. Potter (1996). "Sp4, a member of the Sp1-family of zinc finger transcription factors, is required for normal murine growth, viability, and male fertility." Developmental biology **176**(2): 284-299.

Taketani, S., Y. Adachi, H. Kohno, S. Ikehara, R. Tokunaga and T. Ishii (1998). "Molecular characterization of a newly identified heme-binding protein induced during differentiation of urine erythroleukemia cells." Journal of Biological Chemistry **273**(47): 31388-31394.

Teramitsu, I., L. C. Kudo, S. E. London, D. H. Geschwind and S. A. White (2004). "Parallel FoxP1 and FoxP2 expression in songbird and human brain predicts functional interaction." Journal of Neuroscience **24**(13): 3152-3163.

Thoenes, M., U. Zimmermann, I. Ebermann, M. Ptok, M. A. Lewis, H. Thiele, S. Morlot, M. M. Hess, A. Gal and T. Eisenberger (2015). "OSBPL2 encodes a protein of inner and outer hair cell stereocilia and is mutated in autosomal dominant hearing loss (DFNA67)." Orphanet journal of rare diseases **10**(1): 15.

van Keimpema, M., L. J. Grüneberg, M. Mokry, R. van Boxtel, J. Koster, P. J. Coffer, S. T. Pals and M. Spaargaren (2014). "FOXP1 directly represses transcription of proapoptotic genes and cooperates with NF-κB to promote survival of human B cells." Blood **124**(23): 3431-3440.

Wang, H., Z. Yan, S. Yang, J. Cai, H. Robinson and H. Ke (2008). "Kinetic and structural studies of phosphodiesterase-8A and implication on the inhibitor selectivity." Biochemistry **47**(48): 12760.

Wilson, D. B. and M. P. Wilson (1992). "Identification and subcellular localization of human rab5b, a new member of the ras-related superfamily of GTPases." Journal of Clinical Investigation **89**(3): 996-1005.

XIN, X., R. DAY, W. DONG, Y. LEI and L. D. FRICKER (1998). "Identification of mouse CPX-2, a novel member of the metallocarboxypeptidase gene family: cDNA cloning, mRNA distribution, and protein expression and characterization." DNA and cell biology **17**(10): 897-909.

Xing, G., J. Yao, B. Wu, T. Liu, Q. Wei, C. Liu, Y. Lu, Z. Chen, H. Zheng and X. Yang (2014). "Identification of OSBPL2 as a novel candidate gene for progressive nonsyndromic hearing loss by whole-exome sequencing." Genetics in Medicine **17**(3): 210-218.

Yamazaki, Y., C. Schonherr, G. K. Varshney, M. Dogru, B. Hallberg and R. H. Palmer (2013). "Goliath family E3 ligases regulate the recycling endosome pathway via VAMP3 ubiquitylation." EMBO J **32**(4): 524-537.

Zhu, Q., M. Yamakuchi, S. Ture, M. de la Luz Garcia-Hernandez, K. A. Ko, K. L. Modjeski, M. B. LoMonaco, A. D. Johnson, C. J. O’Donnell, Y. Takai, C. N. Morrell and C. J. Lowenstein (2014). "Syntaxin-binding protein STXBP5 inhibits endothelial exocytosis and promotes platelet secretion." Journal of Clinical Investigation **124**(10): 4503-4516.

Zylka, M. J. and S. M. Reppert (1999). "Discovery of a putative heme-binding protein family (SOUL/HBP) by two-tissue suppression subtractive hybridization and database searches." Molecular brain research **74**(1): 175-181.
