# Supplementary material for: Subcellular analysis of pigeon hair cells implicates vesicular trafficking in cuticulosome formation and maintenance
Source: eLife. 2017 Nov 15;6:e29959. doi: 10.7554/eLife.29959 (PMC5699870; doi:10.7554/eLife.29959)
Supplement: Figure 5—source data 3. — This table shows all GO terms (cellular component) that were significantly enriched in genes that were upregulated in cuticulosome positive hair cells (>3 fold). [file elife-29959-fig5-data3.docx]

| **GO Term**  **(cellular component)** | **GO Accession Number** | **Fold Enrichment** | **P-value** |
| --- | --- | --- | --- |
| extracellular vesicle | GO:1903561 | 1.68 | 2.77E^-02^ |
| extracellular organelle | GO:0043230 | 1.68 | 3.01E^-02^ |
| extracellular exosome | GO:0070062 | 1.66 | 4.35E^-02^ |
| extracellular region part | GO:0044421 | 1.50 | 4.84E^-02^ |
| cytoplasmic part | GO:0044444 | 1.41 | 6.39E^-05^ |
| organelle | GO:0043226 | 1.36 | 1.13E^-10^ |
| membrane-bounded organelle | GO:0043227 | 1.36 | 7.35E^-09^ |
| intracellular membrane-bounded organelle | GO:0043231 | 1.35 | 1.42E^-05^ |
| intracellular organelle | GO:0043229 | 1.33 | 9.25E^-07^ |

**Figure 5- source data 3.** **GO enrichment analysis for “cellular component”.** This table shows all GO terms (cellular component) that were significantly enriched in genes that were upregulated in cuticulosome positive hair cells (>3-fold).
