# Supplementary material for: Subcellular analysis of pigeon hair cells implicates vesicular trafficking in cuticulosome formation and maintenance
Source: eLife. 2017 Nov 15;6:e29959. doi: 10.7554/eLife.29959 (PMC5699870; doi:10.7554/eLife.29959)
Supplement: Figure 5—source data 4. — This table shows all GO terms (molecular function) that were significantly enriched in genes that were upregulated in cuticulosome positive hair cells (>3 fold). [file elife-29959-fig5-data4.docx]

| **GO Term**  **(molecular function)** | **GO Accession Number** | **Fold Enrichment** | **P-value** |
| --- | --- | --- | --- |
| enzyme binding | GO:0019899 | 1.88 | 8.46E^-03^ |
| [hydrolase activity](http://amigo.geneontology.org/amigo/term/GO:0016787) | GO:0016787 | 1.72 | 3.27E^-02^ |
| [metal ion binding](http://amigo.geneontology.org/amigo/term/GO:0046872) | GO:0046872 | 1.69 | 1.55E^-03^ |
| [cation binding](http://amigo.geneontology.org/amigo/term/GO:0043169) | GO:0043169 | 1.68 | 1.35E^-03^ |
| [ion binding](http://amigo.geneontology.org/amigo/term/GO:0043167) | GO:0043167 | 1.58 | 3.26E^-05^ |
| [catalytic activity](http://amigo.geneontology.org/amigo/term/GO:0003824) | GO:0003824 | 1.56 | 2.37E^-05^ |
| protein binding | GO:0005515 | 1.42 | 1.04E^-05^ |
| binding | GO:0005488 | 1.33 | 1.89E^-09^ |

**Figure 5- source data 4.** **GO enrichment analysis for “molecular function”.** This table shows all GO terms (molecular function) that were significantly enriched in genes that were upregulated in cuticulosome positive hair cells (>3-fold).
