# Supplementary material for: Subcellular analysis of pigeon hair cells implicates vesicular trafficking in cuticulosome formation and maintenance
Source: eLife. 2017 Nov 15;6:e29959. doi: 10.7554/eLife.29959 (PMC5699870; doi:10.7554/eLife.29959)
Supplement: Transparent reporting form [file elife-29959-transrepform.docx]

***eLife’s* transparent reporting form**

We encourage authors to provide detailed information *within their submission* to facilitate the interpretation and replication of experiments. Authors can upload supporting documentation to indicate the use of appropriate reporting guidelines for health-related research (see [EQUATOR Network](http://www.equator-network.org/%20)), life science research (see the [BioSharing Information Resource](https://biosharing.org/)), or the [ARRIVE guidelines](http://www.plosbiology.org/article/info:doi/10.1371/journal.pbio.1000412) for reporting work involving animal research. Where applicable, authors should refer to any relevant reporting standards documents in this form.

If you have any questions, please consult our Journal Policies and/or contact us: [editorial@elifesciences.org](mailto:editorial@elifesciences.org).

**Sample-size estimation**

- You should state whether an appropriate sample size was computed when the study was being designed
- You should state the statistical method of sample size computation and any required assumptions
- If no explicit power analysis was used, you should describe how you decided what sample (replicate) size (number) to use

Please outline where this information can be found within the submission (e.g., sections or figure legends), or explain why this information doesn’t apply to your submission:

For our transcriptomic studies we employed a total of 9 biological replicates, each consisting of 30 cuticulosome positive cells, or 30 cuticulosome negative cells. In selecting a n=9 we drew on the published literature on the subject (See (Ching, Huang et al. 2014). Ching and colleagues have reported that the primary determinant of power for RNA-seq experiments is sample size, but is also influence by read depth. Their analysis of 6 published studies showed that higher power is achieved as the number of samples increases, however, beyond a certain number the gain is negligible. Ching et al recommended that n>5, and showed that little power is gained from exceeding a n=10 in a pair experimental design. Their power estimates further revealed that read depths should should exceed 20 million reads per a sample to achieve maximal power - in our case we average 42 million reads per a sample. This is stated in the results of our manuscript.

**Replicates**

- You should report how often each experiment was performed
- You should include a definition of biological versus technical replication
- The data obtained should be provided and sufficient information should be provided to indicate the number of independent biological and/or technical replicates
- If you encountered any outliers, you should describe how these were handled
- Criteria for exclusion/inclusion of data should be clearly stated
- High-throughput sequence data should be uploaded before submission, with a private link for reviewers provided (these are available from both GEO and ArrayExpress)

Please outline where this information can be found within the submission (e.g., sections or figure legends), or explain why this information doesn’t apply to your submission:

We define a biological replicate as a separate biological entity, in contrast to a technical replicate which simply involves multiple measurements of the same sample. This is stated in the methods. In all instances we have provided n numbers which indicate the number of birds analysed (i.e. biological replicates), often coupled with the number of cells from those birds that have been assessed. This is stated in the methods. We did not exclude any outliers, however, for our histological experiments we did exclude samples where the tissue was damaged during sample preparation. Where experiments have been replicated multiple times this is stated in the figure legend.

**Statistical reporting**

- Statistical analysis methods should be described and justified
- Raw data should be presented in figures whenever informative to do so (typically when N per group is less than 10)
- For each experiment, you should identify the statistical tests used, exact values of N, definitions of center, methods of multiple test correction, and dispersion and precision measures (e.g., mean, median, SD, SEM, confidence intervals; and, for the major substantive results, a measure of effect size (e.g., Pearson's r, Cohen's d)
- Report exact p-values wherever possible alongside the summary statistics and 95% confidence intervals. These should be reported for all key questions and not only when the p-value is less than 0.05.

Please outline where this information can be found within the submission (e.g., sections or figure legends), or explain why this information doesn’t apply to your submission:

For analysis of our RNA seq data we employed DEseq2, which calls differentially expressed genes within datasets and incorporates a procedure for multiple testing (Benjamini and Hochberg). This is stated in the results and methods. All graphs show individual values with dots, and the error bars are defined in the figure legend. Exact P values for our gene ontogeny analysis and transcriptomics experiments is shown in Supplementary Tables 3,4, 5 and 6.

(For large datasets, or papers with a very large number of statistical tests, you may upload a single table file with tests, Ns, etc., with reference to sections in the manuscript.)

**Group allocation**

- Indicate how samples were allocated into experimental groups (in the case of clinical studies, please specify allocation to treatment method); if randomization was used, please also state if restricted randomization was applied
- Indicate if masking was used during group allocation, data collection and/or data analysis

Please outline where this information can be found within the submission (e.g., sections or figure legends), or explain why this information doesn’t apply to your submission:

N/A

**Additional data files (“source data”)**

- We encourage you to upload relevant additional data files, such as numerical data that are represented as a graph in a figure, or as a summary table
- Where provided, these should be in the most useful format, and they can be uploaded as “Source data” files linked to a main figure or table
- Include model definition files including the full list of parameters used
- Include code used for data analysis (e.g., R, MatLab)
- Avoid stating that data files are “available upon request”

Please indicate the figures or tables for which source data files have been provided:

Original transcriptomics data has been uploaded to NCBI Gene Expression Omnibus. It can be accessed here:

<https://www.ncbi.nlm.nih.gov/geo/query/acc.cgi?acc=GSE100823>

With the secure code: edgrggswrbsfhir

Ching, T., S. Huang and L. X. Garmire (2014). "Power analysis and sample size estimation for RNA-Seq differential expression." RNA **20**(11): 1684-1696.
